# Pleural effusions are associated with adverse outcomes after cardiac surgery: a propensity-matched analysis

**DOI:** 10.1186/s13019-022-02050-y

**Published:** 2022-12-07

**Authors:** Fridtjof Schiefenhövel, Akira-Sebastian Poncette, Edward M. Boyle, Christian von Heymann, Mario Menk, Gerald Vorderwülbecke, Herko Grubitzsch, Sascha Treskatsch, Felix Balzer

**Affiliations:** 1 Charité – Universitätsmedizin Berlin, corporate Member of Freie Universität and Humboldt-Universität zu Berlin, Department of Anesthesiology and Intensive Care Medicine, Charitéplatz 1, 10117 Berlin, Germany; 2 Charité – Universitätsmedizin Berlin, corporate Member of Freie Universität and Humboldt-Universität zu Berlin, Institute of Medical Informatics, Charitéplatz 1, 10117 Berlin, Germany; 3 Department of Cardiothoracic Surgery, St. Charles Medical Center, Bend, OR USA; 4 Department of Anesthesia, Intensive Care Medicine, Emergency Medicine and Pain Therapy, Vivantes Klinikum im Friedrichshain, Berlin, Germany; 5 Charité – Universitätsmedizin Berlin, corporate Member of Freie Universität and Humboldt-Universität zu Berlin, Department of Cardiovascular Surgery, Augustenburger Platz 1, 13353 Berlin, Germany; 6 Charité – Universitätsmedizin Berlin, corporate Member of Freie Universität and Humboldt-Universität zu Berlin, Department of Anesthesiology and Intensive Care Medicine, Hindenburgdamm 30, 12203 Berlin, Germany; 7 Klinikum rechts der Isar, Technical University of Munich, School of Medicine, Department of Anaesthesiology and Intensive Care, Munich, Germany; 8 Klinikum rechts der Isar, Technical University of Munich, School of Medicine, Institute of Artificial Intelligence and Informatics in Medicine, Munich, Germany

**Keywords:** Pleural effusions, Cardiac surgery, Mortality, Outcomes, Postoperative, Intensive care unit, Intensive care medicine

## Abstract

**Background:**

Pleural effusions commonly occur in patients recovering from cardiac surgery; however, the impact on outcomes is not well characterized. The purpose of this study is to characterize the clinical outcomes of cardiac surgery patients with pleural effusion.

**Methods:**

All patients undergoing cardiac surgery between 2006 and 2019 at a tertiary care university hospital were included in this observational, cross-sectional analysis using propensity matching.

**Results:**

Of 11,037 patients that underwent cardiac surgery during the study period, 6461 (58.5%) had no pleural effusion (Group 0), 3322 (30.1%) had pleural effusion only (Group 1), and 1254 (11.4%) required at least one secondary drainage procedure after the index operation (Group 2). After propensity matching, the mortality of patients who underwent secondary drainage procedures was 6.1% higher than in Group 1 (*p* < 0.001). Intensive care unit (ICU) stay was longer for those with pleural effusions (18 [IQR 9–32] days in Group 2, 10 [IQR 6–17] days for Group 1, and 7 [IQR 4–11] days for Group 0, *p* < 0.001). Patients with pleural effusions had a higher incidence of hemodialysis (246 [20.0%] in Group 2, 137 [11.1%] in Group 1, 98 [7.98%] in Group 0), and a longer ventilation time in the ICU (57 [IQR 21.0-224.0] hours in Group 2, 25.0 [IQR 14.0–58.0] hours in Group 1, 16.0 [IQR 10.0–29.0] hours in Group 0).

**Conclusion:**

Pleural effusions, especially those that require a secondary drainage procedure during recovery, are associated with significantly worse outcomes including increased mortality, longer length of stay, and higher complication rates. These insights may be of great interest to scientists, clinicians, and industry leaders alike to foster research into innovative methods for preventing and treating pleural effusions with the aim of improving outcomes for patients recovering from cardiac surgery.

## Background

Pleural effusions occur frequently in patients recovering from cardiac surgery [1]. Of patients undergoing coronary artery bypass grafting or heart valve surgery, between 41% and 89% develop pleural effusions in the first 7 days after surgery and 10% develop a pleural effusion occupying more than 25% of the hemithorax in the subsequent month [2–5]. Symptomatic patients with pleural effusions may complain of shortness of breath, cough, and chest pain. Thus, pulmonary function in patients with pleural effusion post–internal mammary artery grafting is significantly impaired compared to patients without pleural changes [6]. Clinically significant effusions can delay recovery in the hospital and beyond, and are a critical source of hospital readmissions after discharge [7]. Causes of pleural effusions after cardiac surgery include diaphragm dysfunction, internal mammary artery harvesting (only in internal mammary artery grafting), and other perioperative complications (e.g., sepsis, congestive heart failure, pulmonary embolism, and chylothorax) [8]. However, it is unclear whether pleural effusions are only concomitant symptoms of more complicated cases involving patients with outcome-limiting comorbidities, or if they are themselves associated with impaired outcomes. This study was carried out with the aim of determining the clinical outcomes of pleural effusions in propensity-matched patients during early recovery from cardiac surgery.

## Methods

All patients undergoing cardiac surgery between 2006 and 2019 at a tertiary care university hospital were included in this observational, cross-sectional analysis. With the written consent of the federal data protection officer and the hospital ethics commission, routine clinical data from all eligible patients were extracted from the hospital’s IT system to an anonymized study database. The study was registered on ClinicalTrials.gov (identifier NCT03409055). Cardiac surgery was defined as a documented open procedure on the valves or vessels in proximity to the heart or coronary vessels identified by German OPS codes (5–35 and 5–36, excluding 5–35 A, i.e., minimally invasive valve replacement). Presence of postoperative pleural effusion was derived from the respective ICD-10 codes (J90, J91: pleural effusion; J94.2: hemothorax). We included the codes for hemothorax to capture the full spectrum of patients diagnosed with fluid around their lungs during this study period. By defining the index surgeries for this study using OPS codes, we excluded surgeries e.g. for mediastinitis. The senior physician in the ICU, together with a dedicated, trained administrative staff, completed all coding according to national coding guidelines. Patients were divided into three groups: Group 0 consists of patients with no coding for pleural effusion, Group 1 consists of patients with effusions that either had no postoperative interventions or whose first postoperative intervention was not an isolated secondary drainage procedure, and Group 2 consists of patients with effusions that received an isolated secondary drainage procedure in the ICU as their first intervention following the initial cardiac surgery (“index operation”). An isolated secondary drainage procedure was defined as the coding of a time stamp and at least one OPS code for a secondary drainage procedure throughout the hospital stay (8–152.1: thoracentesis; 8–144: minimal invasive placement of supplemental chest tube; 5–340.0: surgical placement of supplemental chest tube), with no OPS codes indicating any other procedures sharing the same time stamp. In addition to basic patient characteristics, Charlson Comorbidity Index score, surgery type, priority (i.e., elective, urgent, or emergency), duration of surgery (as a marker of its complexity), and post-operative APACHE II admission scores were assessed in order to characterize the study population and to identify possible confounders. Pre-existing medical conditions were derived from ICD-10 diagnostic codes available from the electronic patient records. Redo surgery for evacuation of pleural effusions was defined as thoracotomy, pericardiotomy, or mediastinotomy.

At the completion of surgery, all patients received at least one commercially available 30 French mediastinal chest tube connected to a vacuum for the initial postoperative blood evacuation after the primary surgery via a subcostal approach. In addition, intraoperative pleural tubes were placed when the pleural space was opened, e.g., harvesting LIMA. In case of significant preoperative pleural effusion, e.g., due to congestive heart failure, these patients also received a pleural drainage via subcostal approach during the index surgery. Those “primary” tubes were established by the cardiac surgeon. In case of significant postoperative pleural effusion, an additional “secondary” thoracentesis and/or chest tube was inserted in the anterior axillary line via mini-thoracotomy by the attending ICU practitioner. All chest tubes were actively drained by nurses as needed. All patients received a combined care pathway in the ICU and Intermediate Care Unite (IMCU). Postoperative medication, including diuretics, was supervised by the attending senior physician of the ICU according to common guidelines.

Primary end points were in-hospital mortality and length of stay; deceased patients were set to missing. Duration of mechanical ventilation in the ICU, incidence of renal replacement therapy (RRT) in patients without a medical history of chronic renal failure, or acute kidney injury (rise of creatinine > 0.3 mg/dl in 48 h during the hospital stay) during the hospital stay were defined as secondary end points.

Descriptive analyses and statistical testing were performed using R (version 3.6.3; R Foundation for Statistical Computing). When normal distribution was ruled out using the Kolmogorov–Smirnov test, results were given as medians and interquartile ranges (IQR), otherwise they were given as mean ± standard deviation (SD). Qualitative observations were characterized by numbers with percentages. Statistical significance among groups was analyzed univariately by the exact nonparametric Kruskal–Wallis test and (pairwise) with the exact Mann–Whitney *U* test. We removed the effect of baseline confounder variables by pairwise next neighbor matching (1:1:1). Pairing was done by using the propensity score method, as described previously by Kurth et al. [9]. We created a propensity score and performed next neighbor matching for the first and second group, followed by an additional propensity score creation and matching for the second and third group, with group order 0, 1, and 2. The following variables, which were judged to present major confounders, were used: age, sex, BMI, type of surgery, duration and priority of surgery, ASA classification, NYHA score, congestive heart failure, Charlson Comorbidity Index score, APACHE 2 score upon ICU admission, coronary heart disease, chronic obstructive pulmonary disease, peripheral vascular disease, diabetes, and chronic renal failure. A two-tailed *p*-value < 0.05 was considered statistically significant. The data sets generated and analyzed during this study are not publicly available due to reasons of data privacy; however, they are available from the corresponding author (FB) upon reasonable request.

## Results

Of 11,037 patients that underwent cardiac surgery during the study period, 4576 (41.5%) developed pleural effusion documented in their records. Of these, 3322 patients (30.1% of all patients) did not require any form of additional pleural drainage procedure (Group 1) and 1254 (11.4% of all patients) had at least one thoracentesis or supplemental chest tube placed during the index hospitalization (Group 2).

A total of 1232 (98.2%) Group 2 patients were able to be matched to patients in the two remaining groups, resulting in a sample of 3692 patients (Table 1). In this cohort, the majority of patients were male (63.4%) and the most common procedure was an elective coronary artery bypass graft (42.4%). The three most common pre-existing medical conditions were coronary heart disease (76.6%), congestive heart failure (66.8%), and diabetes (60.3%).

**Table 1:** Basic patient characteristics

Characteristics	All (*N* = 3692)	Group 0 (*N* = 1228)	Group 1 (*N* = 1232)	Group 2 (*N* = 1232)	Overall *p* value	Patients, *n*
Age, median (IQR)	73.0 (66.0–77.0)	72.0 (66.8–77.0)	72.0 (66.0–77.0)	73.0 (66.0–78.0)	0.609	3692
Sex, female, *n* (%)	1351 (36.6)	444 (36.2)	455 (36.9)	452 (36.7)	0.920	3692
BMI, median (IQR)	26.8 (24.0–30.4)	27.4 (24.7–30.8)	27.2 (24.2–30.7)	25.7 (23.0–29.4)	< 0.001	2395
Type of surgery, *n* (%)					0.713	3692
Coronary artery bypass graft	1567 (42.4)	541 (44.1)	515 (41.8)	511 (41.5)		
Combined	733 (19.9)	238 (19.4)	250 (20.3)	245 (19.9)		
Valve	1392 (37.7)	449 (36.6)	467 (37.9)	476 (38.6)		
Duration of surgery, seconds, median (IQR)	12,000 (9600–14,700)	11,700 (9588–14,400)	12,300 (9900–14,700)	12,000 (9900–15,000)	0.002	2941
Priority of surgery, *n* (%)					0.390	3692
Elective	2635 (71.4)	859 (70.0)	891 (72.3)	885 (71.8)		
Urgent/emergency	1057 (28.6)	369 (30.0)	341 (27.7)	347 (28.2)		
ASA, *n* (%)					0.039	3692
1–2	57 (1.54)	20 (1.63)	22 (1.79)	15 (1.22)		
3–5	3018 (81.7)	998 (81.3)	1034 (83.9)	986 (80.0)		
“Missing”	617 (16.7)	210 (17.1)	176 (14.3)	231 (18.8)		
Preexisting medical conditions						
NYHA ≥ 3, *n* (%)	1853 (50.2)	610 (49.7)	622 (50.5)	621 (50.4)	0.906	3692
Congestive heart failure, *n* (%)	2468 (66.8)	814 (66.3)	824 (66.9)	830 (67.4)	0.849	3692
Charlson comorbidity Index, median (IQR)	6.00 (5.00–8.00)	6.00 (5.00–8.00)	6.00 (5.00–8.00)	6.00 (5.00–8.00)	0.371	3692
APACHE II, median (IQR)	20.0 (15.0–27.0)	20.0 (15.0–27.0)	21.0 (15.0–28.0)	20.0 (15.0–26.0)	0.351	3692
Coronary heart disease, *n* (%)	2828 (76.6)	948 (77.2)	939 (76.2)	941 (76.4)	0.827	3692
Chronic obstructive pulmonary disease, *n* (%)	799 (21.6)	258 (21.0)	268 (21.8)	273 (22.2)	0.782	3692
Peripheral vascular disease, *n* (%)	622 (16.8)	214 (17.4)	201 (16.3)	207 (16.8)	0.761	3692
Diabetes, *n* (%)	2226 (60.3)	743 (60.5)	740 (60.1)	743 (60.3)	0.975	3692
Chronic renal failure, *n* (%)	1395 (37.8)	474 (38.6)	455 (36.9)	466 (37.8)	0.695	3692

Group 0: no pleural effusion. Group 1: pleural effusion not requiring an intervention. Group 2: pleural effusion requiring an intervention

Patients with pleural effusions requiring secondary drainage procedures had a higher overall mortality compared with the other two groups (190 [15.4%] in Group 2, 114 [9.3%] in Group 1, and 152 [12.4%] in Group 0, *p* < 0.001; Table 2). Median ICU/IMCU stay was longer for those with pleural effusions (18 [IQR 9–32] days in Group 2, 10 [IQR 6–17] days in Group 1, and 7 [IQR 4–11] days in Group 0, *p* < 0.001). Patients with pleural effusions had a longer median length of stay in hospital (27 [IQR 17–46] days in Group 2, 16 [IQR 11–27] days in Group 1, and 13 [IQR 9–20] days in Group 0, *p* < 0.001). Patients with pleural effusions had a higher incidence of hemodialysis (246 [20.0%] in Group 2, 137 [11.1%] in Group 1, and 98 [7.98%] in Group 0) and a longer ventilation time in the ICU (57 [IQR 21.0–224.0] hours in Group 2, 25.0 [IQR 14.0–58.0] hours in Group 1, and 16.0 [IQR 10.0–29.0] hours in Group 0).

**Table 2:** Outcomes of patients with pleural effusions

Outcomes	All patients (*N* = 3692)	Group 0 (*N* = 1228)	Group 1 (*N* = 1232)	Group 2 (*N* = 1232)	Overall *p* value	Patients, *n*
Mortality (in-hospital), *n* (%)	456 (12.4)	152 (12.4)	114 (9.3)	190 (15.4)	< 0.001	3692
Length of stay in hospital (days), median (IQR)	17.0 (11.0–30.0)	13.0 (9.0–20.0)	16.0 (11.0–27.0)	27.0 (17.0–46.0)	< 0.001	3236
Length of stay in ICU/IMCU (days), median (IQR)	10.0 (6.00–20.0)	7.00 (4.00–11.0)	10.0 (6.00–17.0)	18.0 (9.00–32.0)	< 0.001	3236
Duration of ventilation (hours), median (IQR)	25.0 (13.0–75.0)	16.0 (10.0–29.0)	25.0 (14.0–58.0)	57.0 (21.0-224.0)	< 0.001	3196
AKI^a^, *n* (%)	2752 (74.5)	793 (64.6)	922 (74.8)	1037 (84.2)	< 0.001	3692
RRT^b^, *n* (%)	481 (13.0)	98 (7.98)	137 (11.1)	246 (20.0)	< 0.001	3692
Red blood cell transfusion (units), median (IQR)	4.00 (1.00–8.00)	2.00 (0.00–5.00)	3.00 (1.00–7.00)	6.00 (3.00–13.0)	< 0.001	3692
Hemothorax, *n* (%)	168 (4.55)	37 (3.01)	76 (6.17)	55 (4.46)	0.001	3692
Pleural drainage, *n* (%)	1592 (43.1)	0 (0.00)	93 (0.1)	1499 (94.2)	< 0.001	3692
Redo surgery, *n* (%)	293 (7.94)	85 (6.92)	152 (12.3)	56 (4.55)	< 0.001	3692

Group 0: no pleural effusion. Group 1: pleural effusion not requiring an intervention. Group 2: pleural effusion requiring an intervention

^a^AKI: acute kidney injury defined as increase in serum creatinine of ≥ 0.3 mg/dl within 48 h.

^b^RRT: renal replacement procedure (continuous or discontinuous) in patients who did not have an ICD code of chronic kidney disease.

## Discussion

### Principal findings

This observational, cross-sectional analysis demonstrates that pleural effusions after cardiac surgery occurred in nearly half of all patients undergoing cardiac surgery at the study site. In addition, the occurrence of postoperative “secondary” pleural effusions may have a more important clinical impact than previously assumed. Pleural effusions were associated with an increased mortality and longer stay in hospital. These results suggest that poor outcomes in patients with pleural effusion after cardiac surgery are not only attributable to the presence of preoperative comorbidities or surgical factors, but also to the pleural effusion itself.

### Impact of pleural effusions on clinical outcome after cardiac surgery

Of all patients that underwent cardiac surgery during the study period, 11.4% needed an additional drainage intervention (Group 2). This incidence is slightly higher compared to prior studies; for example, Light et al. found that 9.7% of 389 patients required thoracentesis, while Labidi et al. evaluated 2892 patients, of which 6.6% required pleural drainage [1, 2]. In contrast to a previous study in which pleural effusions were said to play a minor role, our findings indicate that these effusions are more than an incidental finding in patients recovering from cardiac surgery [2]. Part of this discrepancy may be due to the definition of pleural effusions after cardiac surgery. When solely assessing chest X-rays to diagnose pleural effusion, the incidence is as high as 63–89% [10]. These values may be high because of overdiagnosis of pleural effusion due to the inclusion of small, clinically insignificant effusions noted only on X-ray images; however, due to overdiagnosis, the impact on outcomes may be underestimated. A strength of our study is that only clinically significant effusions and drainage interventions documented by the clinicians were included in the analysis, which enabled us to more accurately analyze the clinical outcome.

Since the impact of these effusions on recovery is not well characterized or even stated to be clinically insignificant in the current literature, it is not possible to deduce if additional drainage interventions improve clinical outcome. However, these data suggest that patients requiring a secondary pleural drainage procedure should be identified as high-risk patients for poor outcomes. Pleural effusion in these patients may be an independent risk factor for poor outcome.

Interestingly, in group 2 the rates for AKI and RRT were much higher than compared to group 1 (9.4% and 8.9%, respectively). Considering the cardiorenal syndrome, this could be explained by the fact that AKI and the need for RRT could be a surrogate for a worse outcome [11].

### Potential impact of pleural effusions on economic outcome after cardiac surgery

The aging population, expensive medical treatments, and the rise of chronic diseases are forcing national healthcare institutions to look for more effective healthcare delivery systems [12]. A potential solution may be value-based healthcare, where healthcare providers are rewarded for the quality of clinical outcomes, rather than for the volume of services they provide [13, 14]. In this context, pleural effusions in post–cardiac surgery patients may represent a potentially preventable complication where a marked increase in healthcare resource utilization is required. Successful prevention can not only improve the clinical outcome but may also reduce the economic burden on the healthcare system. Patients who require additional pleural drainage interventions (Group 2) stay in the ICU/IMCU three times longer and have more complications, most likely resulting in higher costs for these patients than for patients without a pleural effusion. The overall high length of stay can in part be explained by the German Diagnosis-related group (DRG) system as well as the fact that patients were directly transferred back to the general ward of the referring cardiology department.

### Prevention of pleural effusions after cardiac surgery

Based on these data, steps should be taken to prevent large pleural effusions that require additional chest tubes. Preoperatively, adequate guideline-based heart failure medication should be a priority; postoperatively, fluid intake should be limited and managed by targeted hemodynamic therapy [15, 16]. Considering that a majority of studies examining cytology point to these effusions discovered early in recovery as exudative, inflammatory, and bloody, the data suggest that remediation efforts might best be focused on sources of local inflammation around the heart and lungs during recovery. However, recent efforts at limiting inflammation with powerful anti-inflammatory drugs have all been disappointing [17, 18]. Most of these have been in the context of studies to prevent post-pericardiotomy syndrome, of which pleural effusion is one of the findings [19].

Focusing on efforts to prevent inadequate evacuation of blood by chest tubes may be a more promising strategy, and should be differentiated from draining serous effusion [20]. In a recent study, the use of multiple mediastinal chest tubes after cardiac surgery showed no advantage over a single chest tube with regard to redo surgery, bleeding, or tamponade, but the impact on the incidence of pleural effusion remained unclear [21]. However, the use of an additional pleural drain, which remained in place for up to 5 days after surgery, reduced the incidence of left pleural effusion [22], while early removal of chest tubes was associated with an increased risk of postoperative pleural effusions [23]. These results underscore the importance of effective pleural drainage after cardiac surgery.

Interestingly, active clearance of chest tubes by ICU nurses at the bedside can prevent chest tube occlusion [24, 25]. Similarly, in a study by Baribeau et al., a protocol including active clearance of chest tubes reduced the pleural intervention rate by 63% [26]. In a protocol utilizing active chest tube clearance technology in the first 24 h after cardiac surgery, pleural effusions were reduced by 47%, suggesting that preventing blood retention by optimizing tube patency may help prevent post-operative pleural effusions [27]. However, in an additional study of active clearance, which uses a tube with an intraluminal magnetic clearance device to break up and clear chest tube clots, the method was associated with a lower rate of redo surgery, but not with a lower incidence of pleural effusion [28]. The role of active clearance on an as-needed basis—using a strict protocol or even a specific technology—versus passive clearance needs to be further investigated.

Our study results suggest that pleural effusions in patients after cardiac surgery have an independent effect on clinical outcome. Efforts should be made to detect insufficient pleural drainage as early as possible (e.g., by daily pleural sonography [29]) and optimize pleural drainage strategies in clinical routine. Novel innovative chest tubes with an active clearing mechanism should be further investigated in the context of pleural effusions.

### Limitations

This study demonstrates that pleural effusions after cardiac surgery, which in the past were often considered a trivial occurrence during recovery, have a significant impact on outcomes and mortality. There are, however, several limitations to the analysis.

First, we used retrospective data from our clinical database. This means that the validity of the diagnostic codes remains uncertain to some extent and that the quality of the data may be impaired by retrospective collection. However, several open-access health datasets (e.g., MIMIC database) have effectively been used for retrospective clinical research [30, 31]. In particular, the secondary analysis of electronic health records plays an important role in cardiovascular research [32]. In the future, these retrospective analyses may foster rapid hypothesis testing prior to costly clinical trials.

Second, neither a standard definition for pleural effusion nor a standard protocol for thoracentesis or chest tube use was used at the time of the study, leaving it to the clinical team to decide the following: (1) when a pleural effusion was rated as clinically relevant, (2) whether to intervene with additional drainage procedures, and (3) whether both diagnosis and procedure were coded. The decision of the clinical team was based on the clinical presentation of the patients (i.e., chest tube output, catecholamine dosage) as well as radiology imaging (i.e., X-ray, CT scan, sonography). This may be a better method to diagnose pleural effusions than a sole chest X-ray for the presence or absence of pleural fluid [2]. Therefore, we assumed that if a patient was coded to have a pleural effusion or additional chest tube, then it was clinically more significant than just an incidental finding on a chest X-ray.

Third, while the propensity scoring may have controlled some of these biases, it is likely that not all confounders contributing to the development of pleural effusions have been controlled. For example, since we did not exclude patients with preoperative pleural effusion, pneumonia, parapneumonic effusions/empyema, mediastinitis or perioperative myocardial infarction these patients would have to be considered as potential confounders. As an academic tertiary hospital, our department is overall providing healthcare to patients with high morbidity. To further enhance comparability of the subgroups, a surgical risk score (e.g., Euroscore) would be beneficial in future studies.

Fourth, in this study, patients were not followed up after hospital discharge. In the series by Labidi et al., 21% of patients who had an initial pleural drainage required a subsequent pleural drainage after discharge [1]. Pleural effusions contribute significantly to the need for readmission after discharge [33]. It is likely, therefore, that our analysis underestimates the impact of pleural effusions on recovery after discharge.

Finally, it is unclear from this study whether there is a way to improve outcomes by treating pleural effusions early or preventing them, if the heart failure medication was insufficient in the first place, or if the acute treatment of the pleural effusion led to increased mortality. This study provides a foundation for prospective studies to evaluate protocols or methods to reduce pleural effusions with the goal of improving outcomes.

## Conclusion

This study demonstrates an association of patients diagnosed with pleural effusions after cardiac surgery with poor clinical outcomes, seemingly independent of the complexity of the surgery and comorbidities such as heart insufficiency. Efforts should be made in prevention, early diagnosis, and consistent treatment of pleural effusions, even if this entails additional costs. Novel pocket-size point-of-care ultrasound devices may support rapid bedside diagnosis and follow-up. This study should motivate researchers to evaluate protocols and innovative methods for preventing pleural effusions with the aim of improving outcomes for patients recovering from cardiac surgery.

## Data Availability

The data sets generated and analyzed during this study are not publicly available due to reasons of data privacy; however, they are available from the corresponding author (FB) upon reasonable request.

## Abbreviations

AKI: Acute kidney injury
ICU: Intensive care unit
IMCU: Intermediate care unit
RRT: Renal replacement therapie
